# Evaluation of allelic alterations in short tandem repeats in papillary thyroid cancer

**DOI:** 10.1002/mgg3.1164

**Published:** 2020-02-11

**Authors:** Zhen Dang, Lu Li, Xia Kong, Guoan Zhang, Qi Liu, Haibin Li, Liang Li, Renya Zhang, Wen Cui, Yequan Wang

**Affiliations:** ^1^ Institute of Forensic Medicine and Laboratory Medicine Jining Medical University Forensic Science Center of Jining Medical University Jining Shandong PR China; ^2^ Department of Pathology Affiliated Hospital of Jining Medical University Jining Shandong PR China; ^3^ Department of Pharmacy The First People' s Hospital Affiliated to Jining Medical University Jining Shandong PR China; ^4^ Department of Pathology The First People' s Hospital Affiliated to Jining Medical University Jining Shandong PR China

**Keywords:** laser capture microdissection, mutation, papillary thyroid cancer, short tandem repeats

## Abstract

**Background:**

Malignant tissue samples may be the only source of biological material for forensic investigations, including individual identification or paternity testing; however, such samples may lead to uncertainties due to frequent genomic aberrations associated with tumors, including alterations of the short tandem repeat (STR) loci used for forensic casework.

**Methods:**

Short tandem repeat loci routinely used in forensic analysis (*n* = 23) were analyzed in 68 surgically removed papillary thyroid cancer specimens. Tumor cells and normal stromal cells were separated by laser capture microdissection.

**Results:**

Four kinds of changes were detected between normal and tumor tissues: partial loss of heterozygosity (pLOH), complete loss of heterozygosity, an additional allele, and a new allele not found in normal tissue. These changes were distributed across 20 of the tested STRs, with no mutations in VWA, D16S539, or Penta D. The most frequently affected locus was D2S1338, and the most frequent type of alteration was pLOH. Samples from patients aged 40–59 years exhibited the highest frequencies of STR variation.

**Conclusion:**

Our results suggest that great care should be taken in the evaluation of DNA typing results obtained from malignant tissues, particularly when no normal tissue reference samples are available.

## Graphical abstract

68 papillary thyroid cancer (PTC) tissue samples were performed to evaluate the allelic alterations in short tandem repeats (STR) in malignant tissue as a source for forensic casework. Tumor tissue should only be used in forensic casework with great care and where no other material is available, particularly in tumors with known higher rates of mutation. Three STR loci, VWA, D16S539, and Penta D may select as potential stable STR loci used in PTC tissues for forensic analysis

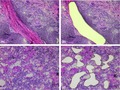

## 1 INTRODUCTION

Papillary thyroid cancer (PTC) is the most common malignancy of the endocrine system, and the incidence rate of PTC has significantly increased in China (Zhang et al., 2013). Although many studies have investigated oncogenic genetic alterations, the molecular mechanisms underlying PTC have yet to be fully elucidated (Kong et al., 2013). Therefore, sensitive markers for the diagnosis of PTC and efficient target genes for the treatment of this disease are required (Kong, Man, Wang, Zhang, & Cui, 2015).

Short tandem repeats (STRs) are highly polymorphic and commonly used for the identification of individuals within populations because of the large number of structured repeat alleles per locus, which are inherited in a Mendelian fashion (Vauhkonen et al., 2004). STR loci are used routinely in forensic DNA studies for the identification of individuals and for establishing paternity (Peloso, Grignani, Rosso, & Previderè, 2003). Many archival pathology specimens can be successfully employed for forensic casework, such as doctor–patient disputes, improper management of post‐operative tissue in hospitals, resulting in misdiagnosis of patients; false tumor tissue information provided by patients to defraud insurance companies, et al; since STRs are stable in the majority of tissues, even post‐mortem (Poetsch et al., 2004). By contrast, the use of tumor tissues for forensic analyses is associated with specific challenges because tumor DNA frequently harbors genetic alterations, not only in defined coding regions but also in repetitive DNA sequences. Four types of allelic alterations in different tumor tissue were reported in the literature, i.e. the occurrence of new alleles in addition to those displayed in the normal tissue (Additional alleles), additional allele refers to the additional allele that appears simultaneously with the original allele in the cancer tissue, such as "9, 10" allele mutation in the normal tissue to "9, 10, 11" in the cancer tissue. The occurrence of new alleles instead of those found in normal tissue (New alleles), a new allele is defined as the disappearance of an original allele, which is replaced by a new allele; for example, the "9, 12" allele in normal tissues is mutated into the "10, 12" allele in cancer tissues (Zhang et al., 2018). Complete loss of heterozygosity (LOH) and partial loss of heterozygosity (pLOH) (Li, Zhao, Fang, Liu, & Li, 2009). Noteworthy, the first three variations mentioned above could lead to STR genotype alteration (STR_GA_). However, the variation of pLOH showed bad heterozygosity balance and did not change the STR genotype.

As there is a high prevalence of PTC in the southwest of Shandong province, we collected and analyzed 68 PTC tumor samples, along with normal stromal cells from adjacent non‐cancerous tissues (Kong et al., 2015, 2013; Zhang et al., 2013). The aim of this study was to detect possible STR alterations in PTC tissue samples and estimate the reliability of such samples as a source of genetic information.

## 2 MATERIALS AND METHODS

### 2.1 Samples

Samples and patient data were obtained and used with the ethical approval of the Medical Ethics Committee of Jining Medical University. Fresh PTC specimens (*n* = 68) and corresponding samples from adjacent normal tissue were obtained with informed consent from the Affiliated Hospital of Jining Medical University, Shandong, PR China. All samples were unambiguously diagnosed by pathological examination.

### 2.2 Preparation of cryosections for laser capture microdissection (LCM)

Tissue samples were sectioned in a cryostat to a thickness of 8 μm; a minimum of three sections per sample were prepared for LCM. Cryosections were placed on membrane‐coated microscope slides (Molecular Machines & Industries, Switzerland) and then immediately fixed in 100% ethanol for 1 min. After staining with hematoxylin‐eosin, slides were allowed to dry under a fume hood for 4–6 min to facilitate evaporation of residual xylene before microdissection. Slides were stored at −20°C for later use.

### 2.3 LCM

PTC tumor cells and normal stromal cells were separated by MMI CellCut (Molecular Machines & Industries). Once cells of interest were identified, the operator directed a low‐power laser beam to melt the membrane coating the microscope slide into the selected cells. When the laser beam was interrupted, and the membrane removed, the cells bonded to it by the laser were selectively lifted off. For each sample, 5 × 10^4^ of each cell type (normal and tumor) were collected.

### 2.4 DNA amplification

DNA samples were amplified using a Huaxia™ Platinum PCR amplification kit (Life Technologies Biotechnology), which included assays for Amelogenin and 23 other loci (D19S433, D5S818, D21S11, D18S51, D6S1043, D3S1358, D13S317, D7S820, D16S539, CSF1PO, Penta D, vWA, D8S1179, TPOX, Penta E, TH01, D12S391, D2S1338, FGA, D2S441, D22S1045, D10S1248, and D1S1656) (Wang et al., 2016). Thermal cycling conditions were those detailed in the kit manufacturer's instructions, and reactions were performed using a GeneAmp PCR system 9700 (Applied Biosystems).

### 2.5 STR genotyping

PCR products were separated by capillary electrophoresis using an ABI 3500 Genetic Analyzer (Applied Biosystems), and raw data were analyzed for genotype assignment with GeneMapper ID‐X system (Applied Biosystems) where analytical and stochastic thresholds were set 50 RFU and 200 RFU separately.

### 2.6 Analysis of STR loci

Positive and negative control were detected concurrently to assure data quality. Only variations that were identical for one sample analyzed in triplicate were included in the analysis. All results from normal and tumor tissues were analyzed in a double‐blind manner and then compared. Three of four types of allelic alterations, Additional alleles, New alleles and LOH, could be discerned according to electrophoretogram of normal/tumor pair. Heterozygosity balance measured as ratio of allele peak heights was calculated for the evaluation of decline tendency of heterozygosity alleles. pLOH was scored if one allele was decreased by >50% in the tumor samples, compared with the same allele in the corresponding normal sample. Then allelic loss, i.e. the ratio of heterozygosity balance in normal/tumor pair was used to compute pLOH, if it is showed by an value of <0.5 or >2.0 (Li et al., 2009).

### 2.7 Statistical analysis

Hardy–Weinberg equilibrium software was used to test the distribution of genotype data for each locus. Genotype frequencies were calculated using PowerStats V12 software. The 95% confidence intervals (CI) for mutation rates were derived based on the binomial distribution and obtained via the website http://statpages.org/confint.html. Statistical comparisons were performed using the SPSS 17.0 software package.

## 3 RESULTS

### 3.1 Types of STR mutations in PTC

**Figure 1:**
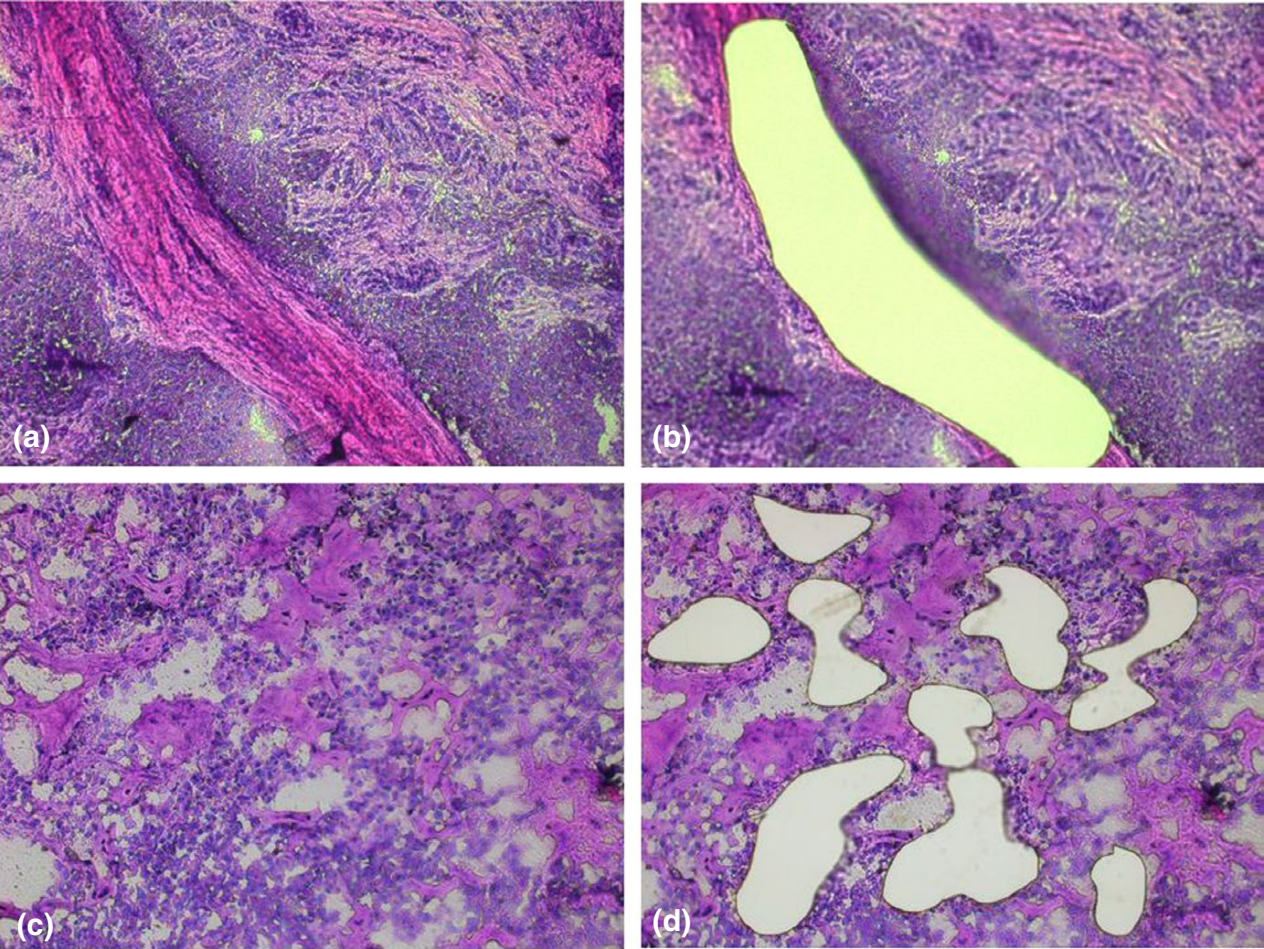
Microscopic images of two PTC tissue samples (×200). (a) Stromal cells before microdissection. (b) Stromal cells after microdissection. (c) Tumor cells before microdissection. (d) Tumor cells after microdissection. PTC, papillary thyroid cancer

**Figure 2:**
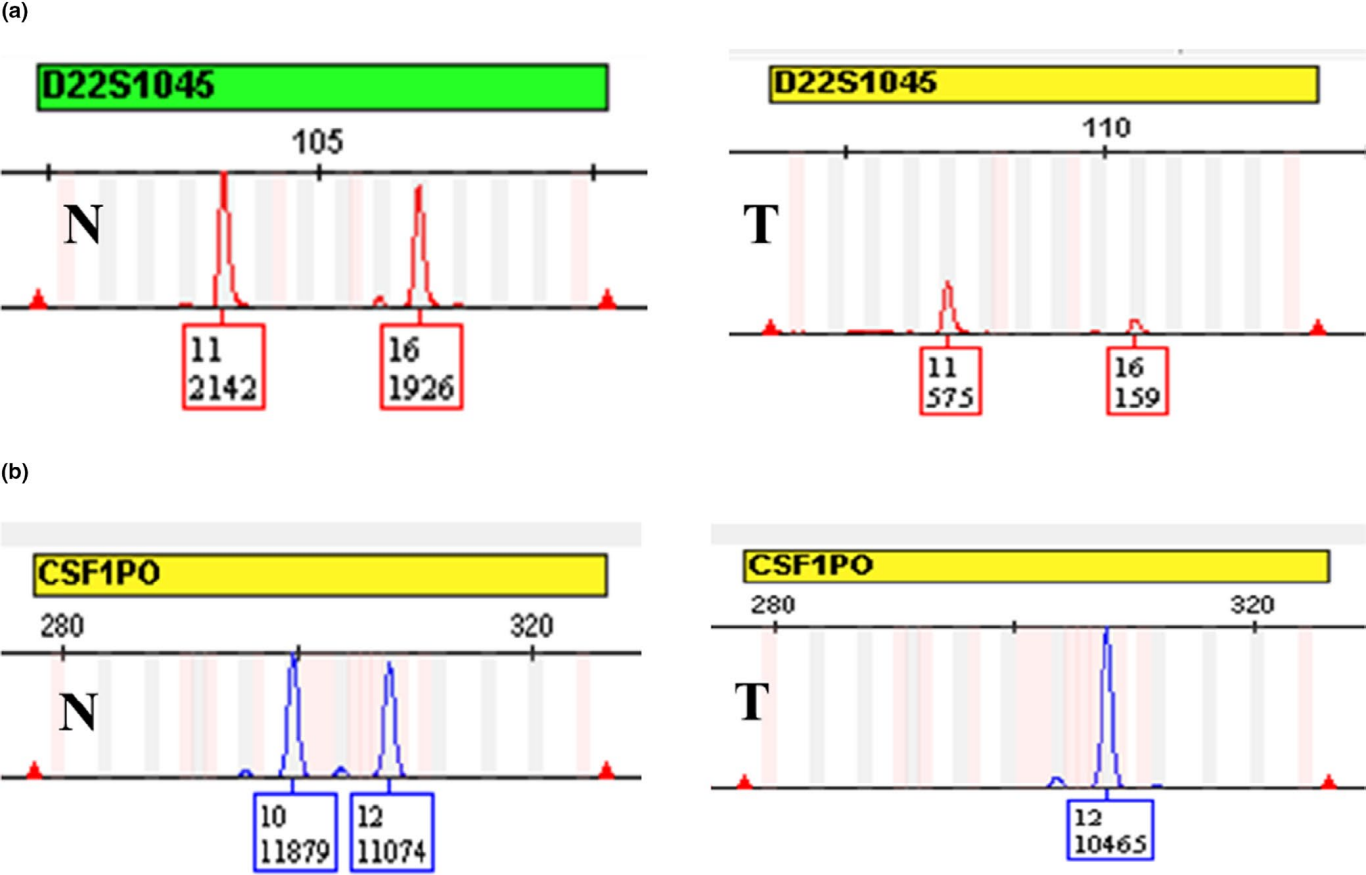
Representative images showing partial and complete loss of heterozygosity genetic alterations in papillary thyroid cancer tissues. (a) Partial loss of allele 16 of D22S1045. (b) Complete loss of allele 10 of CSF1PO. T, tumor cells; N, normal stromal cells

**Figure 3:**
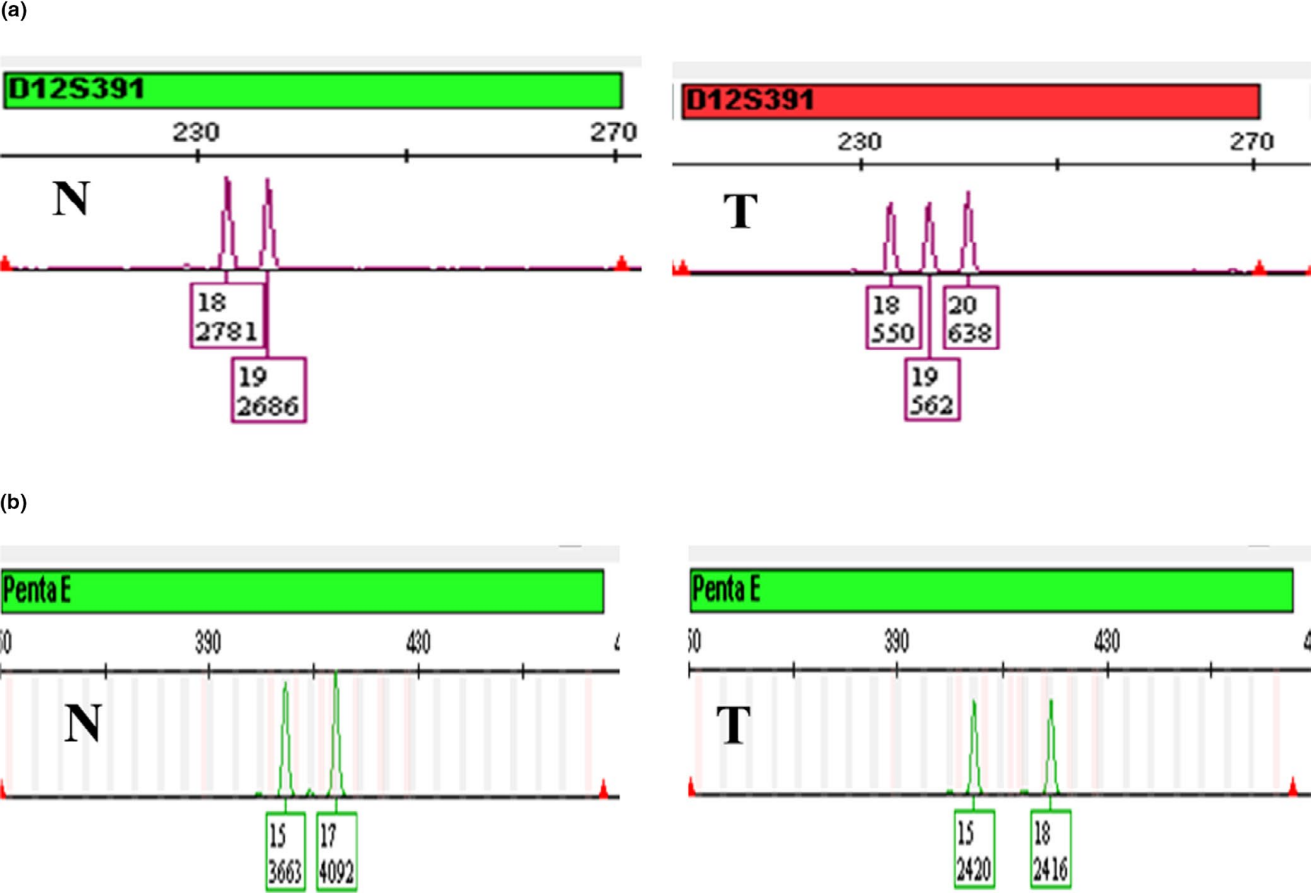
Representative images showing genetic alterations, including additional and new alleles in papillary thyroid cancer tissues. (a) Presence of allele 20, in addition to alleles 18 and 19 of D12S391. (b) Presence of allele 18 instead of allele 17 of Penta E. T, tumor cells; N, normal stromal cells

In this study, each PTC tissue sample was separated into cancer cells and normal stromal cells, from tissue adjacent to the carcinoma, by microdissection (Figure 1). The correct genotypes of positive control samples were acquired and no extra peaks were detected in negative control samples. Four types of allelic alterations were identified in PTC cells in this study: pLOH, LOH (Figure 2), Additional alleles (Figure 3a), and New alleles (Figure 3b).

### 3.2 Frequencies of different autosomal STR mutation types in PTC

The rates of the four mutation types in autosomal STRs in PTC are presented in Table 1. pLOH was the most frequently observed alteration. The same genotypes were present in both normal and tumor tissue specimens in 76.5% of samples, while the remainder had at least one altered locus. Moreover, there were 1,564 loci observed in total, the overall rate of variation detection was 2.17%, and the incidence rate of pLOH, LOH, Additional alleles and New alleles was respectively 1.02%, 0.19%, 0.06%, and 0.90%. In addition, the rate of STR_GA_ was 1.15%. Genetic alterations were present in 20 of the 23 STRs, with no changes detected in VWA, D16S539, or Penta D. D2S1338 was the most frequently affected STR locus (0.26%), and CSF1PO was the second most commonly mutated locus (0.19%) (Table 2).

**Table 1:** The rate of four types of mutation of autosomal STRs in papillary thyroid cancer

Mutation type	No. of mutations	Mutation rate (total no. of loci = 1,564)	95% CI
pLOH (partial loss)	16	0.0102	0.0059–0.0166
LOH (complete loss)	3	0.0019	0.0004–0.0056
Additional alleles	1	0.0006	0.0000–0.0036
New alleles	14	0.0090	0.0049–0.0150
STR_GA_	18	0.0115	0.0068–0.0181

Abbreviations: 95% CI, 95% confidence interval; LOH, loss of heterozygosity; pLOH, partial loss of heterozygosity; STR, short tandem repeat; STR_GA_, STR genotype alteration, including LOH, additional alleles, and new alleles.

**Table 2:** Frequencies of the four mutation types at 23 chromosomal loci in papillary thyroid cancer

Locus	Total mutation rate (%)	Number of mutations (%)				STR_GA_ (%)
		pLOH	LOH	Additional alleles	New alleles	
D3S1358	2 (0.13)	1 (0.06)	0 (0.00)	0 (0.00)	1 (0.06)	1 (0.06)
vWA	0 (0.00)	0 (0.00)	0 (0.00)	0 (0.00)	0 (0.00)	0 (0.00)
D16S539	0 (0.00)	0 (0.00)	0 (0.00)	0 (0.00)	0 (0.00)	0 (0.00)
CSF1PO	3 (0.19)	1 (0.06)	1 (0.06)	0 (0.00)	1 (0.06)	2 (0.13)
TPOX	2 (0.13)	1 (0.06)	0 (0.00)	0 (0.00)	1 (0.06)	1 (0.06)
D8S1179	2 (0.13)	1 (0.06)	0 (0.00)	0 (0.00)	1 (0.06)	1 (0.06)
D21S11	2 (0.13)	1 (0.06)	0 (0.00)	0 (0.00)	1 (0.06)	1 (0.06)
D18S51	1 (0.06)	0 (0.00)	0 (0.00)	0 (0.00)	1 (0.06)	1 (0.06)
Penta E	2 (0.13)	0 (0.00)	0 (0.00)	0 (0.00)	2 (0.13)	2 (0.13)
D2S441	1 (0.06)	0 (0.00)	1 (0.06)	0 (0.00)	0 (0.00)	1 (0.06)
D19S433	1 (0.06)	1 (0.06)	0 (0.00)	0 (0.00)	0 (0.00)	0 (0.00)
TH01	1 (0.06)	1 (0.06)	0 (0.00)	0 (0.00)	0 (0.00)	0 (0.00)
FGA	2 (0.13)	1 (0.06)	0 (0.00)	0 (0.00)	1 (0.06)	1 (0.06)
D22S1045	2 (0.13)	1 (0.06)	1 (0.06)	0 (0.00)	0 (0.00)	1 (0.06)
D5S818	1 (0.06)	0 (0.00)	0 (0.00)	0 (0.00)	1 (0.06)	1 (0.06)
D13S317	2 (0.13)	1 (0.06)	0 (0.00)	0 (0.00)	1 (0.06)	1 (0.06)
D7S820	1 (0.06)	1 (0.06)	0 (0.00)	0 (0.00)	0 (0.00)	0 (0.00)
D6S1043	1 (0.06)	0 (0.00)	0 (0.00)	0 (0.00)	1 (0.06)	1 (0.06)
D10S1248	1 (0.06)	1 (0.06)	0 (0.00)	0 (0.00)	0 (0.00)	0 (0.00)
D1S1656	1 (0.06)	1 (0.06)	0 (0.00)	0 (0.00)	0 (0.00)	0 (0.00)
D12S391	2 (0.13)	0 (0.00)	0 (0.00)	1 (0.06)	1 (0.06)	2 (0.13)
D2S1338	4 (0.26)	3 (0.19)	0 (0.00)	0 (0.00)	1 (0.06)	1 (0.06)
PentaD	0 (0.00)	0 (0.00)	0 (0.00)	0 (0.00)	0 (0.00)	0 (0.00)

Abbreviations: LOH, loss of heterozygosity; pLOH, partial loss of heterozygosity; STR, short tandem repeat; STR_GA_, STR genotype alteration, including LOH, additional alleles, and new alleles. Total no. of Loci = 1,564.

### 3.3 Frequencies of four types of mutation on different chromosomes in PTC

Table 3 shows the rate of the four mutation types at the 23 chromosomal loci in PTC. Markers on chromosome 2, including TPOX, D2S1338, and D2S441, were most frequently affected (0.45%), with those on chromosome 5 (D5S818 and CSF1PO) the second most commonly mutated (0.26%).

**Table 3:** Frequencies of four types of mutation on different chromosomes in papillary thyroid cancer

Chromosome	Locus	STR_GA_ (%)	Total mutation rate (%)
1	D1S1656	0 (0.00)	1 (0.06)
2	TPOX, D2S1338, D2S441	3 (0.19)	7 (0.45)
3	D3S1358	1 (0.06)	2 (0.13)
4	FGA	1 (0.06)	2 (0.13)
5	D5S818, CSF1PO	3 (0.19)	4 (0.26)
6	D6S1043	1 (0.06)	1 (0.06)
7	D7S820	0 (0.00)	1 (0.06)
8	D8S1179	1 (0.06)	2 (0.13)
10	D10S1248	0 (0.00)	1 (0.06)
11	TH01	0 (0.00)	1 (0.06)
12	vWA, D12S391	2 (0.13)	2 (0.13)
13	D13S317	1 (0.06)	2 (0.13)
15	Penta E	2 (0.13)	2 (0.13)
16	D16S539	0 (0.00)	0 (0.00)
18	D18S51	1 (0.06)	1 (0.06)
19	D19S433	0 (0.00)	1 (0.06)
21	Penta D, D21S11	1 (0.06)	2 (0.13)
22	D22S1045	1 (0.06)	2 (0.13)

Abbreviation: STR_GA_, STR genotype alteration, including LOH, additional alleles, and new alleles. Total no. of Loci = 1,564.

### 3.4 Association analysis of STR variations and patient demographic characteristics

Chi‐square tests were used to determine associations between the detection of any STR variant (compared with no STR variant) and patient gender, patient age at surgery/diagnosis, tumor size, and metastasis. The results showed that there were no significant associations between STR variants and patient gender, tumor size, or metastasis; however a significant association was detected between STR variants and patient age at surgery/diagnosis (Table 4). Patients aged 40–59 years exhibited the highest frequency of STR variations (*p* = .0089).

**Table 4:** Association of STR loci variation with clinical and pathological characteristics of patients with PTC

Characteristic	*n*	STR locus variation (*n* = 16)	No STR locus variation (*n* = 52)	*p*‐value
Age				
<40	23	1 (4.4%)	22 (95.6%)	
40–59	37	14 (37.8%)	23 (62.2%)	.0089
60–79	8	1 (12.5%)	7 (87.5%)	
Sex				
Female	48	9 (18.75%)	39 (81.25%)	.2603
Male	20	7 (35.0%)	13 (65.0%)	
Tumor size				
<1 cm	38	10 (26.3%)	28 (73.7%)	.5421
≥1 cm	30	6 (20.0%)	24 (80.0%)	
Metastasis				
Positive	24	5 (20.8%)	19 (79.2%)	.6987
Negative	44	11 (25.0%)	33 (75.0%)	

Abbreviations: PTC, papillary thyroid cancer; STR, short tandem repeat.

## 4 DISCUSSION

In forensic medicine and pathology, sometimes the only source of DNA from a deceased person is a tumor tissue specimen from a pathology laboratory. DNA extracted from such samples can be used to represent individuals in paternity testing or for identification purposes; however, the unstable nature of tumor genomes can be problematic when tumor tissues are used for medico‐legal analyses (Filoglu et al., 2014). In this study, we determined the potential impact of STR instability in PTC on forensic casework.

Clinically resected bulk cancer tissues contain not only cancer cells but also diverse types of stromal cells; for example, fibroblasts, lymphocytes, and vascular endothelial cells (Pai et al., 2002). Such cells can not only affect gene expression pattern profiles, but also preclude accurate analysis of gene expression in cancer cells. Therefore, we used LCM, a sophisticated method for the dissection of tumor cells from whole tumor tissues in experiments to scientifically assess tumor characteristics.(Sugiyama, Sugiyama, Hirai, Akiyama, & Hasumi, 2002; Vandewoestyne, Nieuwerburgh, Hoofstat, & Deforce, 2012).

Four types of allelic alterations were identified in PTC cells. pLOH was the most frequently observed alteration, consistent with a previous study that identified pLOH as the highest frequency mutation in STRs in lung cancer (Zhang et al., 2018). Among the 23 STR loci, the most frequently affected STR locus was at D2S1338. STR_GA_ was detected at the chromosomal loci: 2p, 2q, 3p, 4q, 5q, 6q, 8q, 12p, 13q, 15q, 18q, 21q, and 22q. In addition, the results revealed a significant association between STR variation frequency and patient age at diagnosis, with a significantly higher frequency of STR variants in patients aged 40–59 years (*p* = .0089). Overall, these findings contribute to a better understanding of the genetic mechanisms underlying PTC; however, additional studies will be necessary to explore whether these allelic alterations are involved in the process of carcinogenesis, clinicopathological parameters, or prognosis.

At present, the mechanisms underlying STR mutation remain poorly understood. DNA slippage appears to be widely accepted as the main explanation for STR mutations (Fan & Chu, 2007). In six of 68 samples, altered alleles were simultaneously detected at more than three STR loci, and different types of STR mutations were also detected at the same markers (Table S1); for example, pLOH, LOH, and the creation of a new allele were identified at CSF1PO. We also identified three unchanged STRs (VWA, D16S539, and Penta D) which could potentially be employed in forensic casework as stable STR loci. Therefore, future studies with increased sample size are required to confirm these findings.

We also compared our data with those generated from gynecological cancers and gastrointestinal tumors (Li et al., 2009; Sun et al., 2017). The STR mutation rates of gynecological cancers (5.25%) and gastrointestinal tumors (4.69%) were higher than those detected in PTC samples (1.15%). Our results showed that PTC is associated with lower mutation rates at STRs than other types of tumor, indicating that PTC may have higher value for judicial application.

## 5 CONCLUSION

Based on these results, we suggest that tumor tissue should only be used in forensic casework with great care and where no other material is available, particularly for tumors with known higher rates of mutation. The analysis of additional types of malignancy could be useful to determine which tumors may be employed in forensic casework with minimal risk, such as PTC. Additionally, further studies are necessary to investigate whether these unstable STR loci are involved in the genetic mechanisms underlying PTC etiology.

## CONFLICT OF INTEREST

We declared that we have no conflicts of interest to this work.

## References

[mgg31164-bib-0001] Fan, H. , & Chu, J. Y. (2007). A brief review of short tandem repeat mutation. Genomics Proteomics Bioinformatics, 5, 7–14. 10.1016/S1672-0229(07)60009-6 17572359 PMC5054066

[mgg31164-bib-0002] Filoglu, G. , Bulbul, O. , Rayimoglu, G. , Yediay, F. E. , Zorlu, T. , Ongoren, S. , & Altuncul, H. (2014). Evaluation of reliability on STR typing at leukemic patients used for forensic purposes. Molecular Biology Reports, 41, 3961–3972. 10.1007/s11033-014-3264-9 24562624

[mgg31164-bib-0003] Kong, L. L. , Man, D. M. , Wang, T. , Zhang, G. A. , & Cui, W. (2015). siRNA targeting RBP2 inhibits expression, proliferation, tumorigenicity and invasion in thyroid carcinoma cells. Oncology Letters, 10, 3393–3398. 10.3892/ol.2015.3782 26788140 PMC4665154

[mgg31164-bib-0004] Kong, L. , Zhang, G. , Wang, X. , Zhou, J. , Hou, S. , & Cui, W. (2013). Immunohistochemical expression of RBP2 and LSD1 in papillary thyroid carcinoma. Romanian Journal of Morphology and Embryology, 54, 499–503. 24068396

[mgg31164-bib-0005] Li, C. , Zhao, S. , Fang, J. , Liu, Y. , & Li, L. (2009). Evaluation of reliability of STR typing in human colon carcinomas tissues used for identification purpose. Forensic Science International Genetics Supplement, 2, 8–9. 10.1016/j.fsigss.2009.08.012

[mgg31164-bib-0006] Pai, C. Y. , Hsieh, L. L. , Tsai, C. W. , Chiou, F. S. , Yang, C. H. , & Hsu, B. D. (2002). Allelic alterations at the STR markers in the buccal tissue cells of oral cancer patients and the oral epithelial cells of healthy betel quid‐chewers: An evaluation of forensic applicability. Forensic Science International, 129, 158–167. 10.1016/S0379-0738(02)00205-0 12372686

[mgg31164-bib-0007] Peloso, G. , Grignani, P. , Rosso, R. , & Previderè, C. (2003). Forensic evaluation of tetranucleotide str instability in lung cancer. In: *International Congress Series*.

[mgg31164-bib-0008] Poetsch, M. , Petersmann, A. , Woenckhaus, C. , Protzel, C. , Dittberner, T. , Lignitz, E. , & Kleist, B. (2004). Evaluation of allelic alterations in short tandem repeats in different kinds of solid tumors—Possible pitfalls in forensic casework. Forensic Science International, 145, 1–6. 10.1016/j.forsciint.2004.03.006 15374588

[mgg31164-bib-0009] Sugiyama, Y. , Sugiyama, K. , Hirai, Y. , Akiyama, F. , & Hasumi, K. (2002). Microdissection is essential for gene expression profiling of clinically resected cancer tissues. American Journal of Clinical Pathology, 117, 109–116. 10.1309/G1C8-39MF-99UF-GT2K 11789716

[mgg31164-bib-0010] Sun, L. J. , Li, S. J. , Fu, G. M. , Bai, M. , Dong, C. N. , Yang, B. , … Cong, B. (2017). Mutation analysis of autosomal and X chromosomal STR in gynecologic and breast cancer. Chinese Journal of Forensic Medicine, 32, 350–353.

[mgg31164-bib-0011] Vandewoestyne, M. , Van Nieuwerburgh, F. , Van Hoofstat, D. , & Deforce, D. (2012). Evaluation of three DNA extraction protocols for forensic STR typing after laser capture microdissection. Forensic Science International: Genetics, 6, 258–262. 10.1016/j.fsigen.2011.06.002 21727054

[mgg31164-bib-0012] Vauhkonen, H. , Hedman, M. , Vauhkonen, M. , Kataja, M. , Sipponen, P. , & Sajantila, A. (2004). Evaluation of gastrointestinal cancer tissues as a source of genetic information for forensic investigations by using STRs. Forensic Science International, 139, 159–167. 10.1016/j.forsciint.2003.10.016 15040910

[mgg31164-bib-0013] Wang, Z. , Zhou, D. , Jia, Z. , Li, L. , Wu, W. , Li, C. , & Hou, Y. (2016). Developmental Validation of the Huaxia Platinum System and application in 3 main ethnic groups of China. Scientific Reports, 6, 31075 10.1038/srep31075 27498550 PMC4976323

[mgg31164-bib-0014] Zhang, G. A. , Hou, S. , Han, S. , Zhou, J. , Wang, X. , & Cui, W. (2013). Clinicopathological implications of leptin and leptin receptor expression in papillary thyroid cancer. Oncology Letters, 5, 797–800. 10.3892/ol.2013.1125 23425972 PMC3576217

[mgg31164-bib-0015] Zhang, P. , Zhu, Y. , Li, Y. , Zhu, S. , Ma, R. , Zhao, M. , & Li, J. (2018). Forensic evaluation of STR typing reliability in lung cancer. Legal Medicine, 30, 38–41. 10.1016/j.legalmed.2017.11.004 29154002

