# Supplementary material for: Evaluation of allelic alterations in short tandem repeats in papillary thyroid cancer
Source: Mol Genet Genomic Med. 2020 Feb 11;8(4):e1164. doi: 10.1002/mgg3.1164 (PMC7196467; doi:10.1002/mgg3.1164)
Supplement: Supplementary file 1 [file MGG3-8-e1164-s001.doc]

| Sample | Locus | Alleles  （Normal） | Alleles  (Tumor) | Mutation type |
| --- | --- | --- | --- | --- |
| 1 | D5S818 | 9/11 | 10/11 | New alleles |
|  | D13S317 | 11/12 | 10/12 | New alleles |
|  | D2S1338 | 20/20 | 20/23 | New alleles |
|  | D12S391 | 20/23 | 22/23 | New alleles |
| 2 | D18S51 | 13/15 | 15/17 | New alleles |
|  | D2S441 | 11/12 | 11 | LOH |
|  | FGA | 23/24 | 24/26 | New alleles |
|  | D22S1045 | 11/16 | 16 | LOH |
| 3 | D2S1338 | 19/24 | 19/24 | pLOH |
|  | TPOX | 8/9 | 8/9 | pLOH |
|  | TH01 | 7/9 | 7/9 | pLOH |
| 4 | D10S1248 | 13/14 | 13/14 | pLOH |
|  | D2S1338 | 18/20 | 18/20 | pLOH |
|  | D1S1656 | 15/16 | 15/16 | pLOH |
|  | CSF1PO | 9/12 | 9/12 | pLOH |
| 5 | D8S1179 | 13/14 | 13/14 | pLOH |
|  | D22S1045 | 15/16 | 15/16 | pLOH |
|  | D21S11 | 30/31 | 30/31 | pLOH |
| 6 | D2S441 | 11/12 | 12/14 | New alleles |
|  | D6S1043 | 12/14 | 12/14 | pLOH |
|  | FGA | 22/26 | 22/26 | pLOH |

Table S1 Samples with more than three loci mutated.
